# The Calcified Vasculature in Chronic Kidney Disease Secretes Factors that Inhibit Bone Mineralization

**DOI:** 10.1002/jbm4.10610

**Published:** 2022-03-01

**Authors:** Maria L. Mace, Eva Gravesen, Anders Nordholm, Soeren Egstrand, Marya Morevati, Klaus Olgaard, Ewa Lewin

**Affiliations:** ^1^ Department of Nephrology, Rigshospitalet University of Copenhagen Copenhagen Denmark; ^2^ Department of Pathology, Herlev Hospital University of Copenhagen Copenhagen Denmark; ^3^ Department of Nephrology, Herlev Hospital University of Copenhagen Copenhagen Denmark

**Keywords:** ACTIVIN A, CKD‐MBD, RENAL OSTEODYSTROPHY, TISSUE CROSSTALK, WNT INHIBITORS

## Abstract

Vascular calcification and bone disorder progress simultaneously in chronic kidney disease (CKD). Still, how the complex pathological mechanisms are linked is only sparsely understood. Up to now, the focus has been on the disturbed bone metabolism in developing vascular calcification. However, our group has recently demonstrated that vascular calcification has negative effects on bone formation and mineralization as shown in the bone of normal recipient rats transplanted with the calcified aorta from CKD rats. In the present in vitro study, the hypothesis of a direct crosstalk between the vasculature and bone was examined. Calcified aortas from 5/6 nephrectomized rats and normal aortas from control rats were excised and incubated ex vivo. The calcified aorta secreted large amounts of sclerostin, dickkopf‐1 (Dkk1), and activin A. Both normal and calcified aortas secreted frizzle‐related protein 4 (SFRP4). Aorta rings were co‐incubated with the osteoblast‐like cell line UMR‐106. The calcified aorta strongly inhibited calcium crystal formation in UMR‐106 cells, together with a significant upregulation of the mineralization inhibitors osteopontin and progressive ankylosis protein homolog (ANKH). The strong stimulation of osteopontin was blocked by lithium chloride, indicating involvement of Wnt/β‐catenin signaling. The present in vitro study shows detrimental effects of the calcified aorta on bone cell mineralization. These findings support the hypothesis of an active role of the calcified vasculature in the systemic CKD–mineral and bone disorder (CKD‐MBD), resulting in a pathological vascular–bone tissue crosstalk. © 2022 The Authors. *JBMR Plus* published by Wiley Periodicals LLC on behalf of American Society for Bone and Mineral Research.

## Graphical abstract

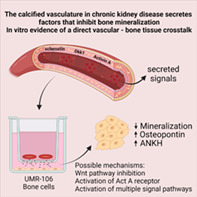

## Introduction

For over three decades, numerous observational studies have reported an association between low bone mineral density (BMD) and the presence of vascular calcification, indicating a mechanistic link between the two pathological processes.^(^ ^1^ ^)^ The progression of the disturbances in the skeletal and cardiovascular system is also linked, as demonstrated in longitudinal cohorts where further development of vascular calcification was accompanied with greater bone loss.^(^ ^2^, ^3^, ^4^ ^)^ Also, the presence of vascular calcification is related to increased fracture risk and vice versa.^(^ ^5^, ^6^, ^7^ ^)^ The phenomenon is especially pronounced in chronic kidney disease–mineral and bone disorder (CKD‐MBD),^(^ ^8^ ^)^ but it is also observed across a wide range of conditions such as aging, diabetes, osteoporosis, and more rare bone diseases.^(^ ^9^, ^10^, ^11^, ^12^, ^13^, ^14^ ^)^ Up to now, the main focus on *the calcification paradox* has been on disturbances in the mineral balance and bone turnover as a causative factor in the development and progression of vascular calcification.^(^ ^15^ ^)^ However, we propose that vascular calcification impairs bone metabolism, generating a pathological vascular–bone tissue crosstalk in CKD.^(^ ^16^ ^)^

CKD patients suffers from a complex disturbance in their mineral and bone balance, which is believed to play a fundamental role in the pathogenesis of vascular calcification in CKD.^(^ ^15^ ^)^ Still, it is important to keep in mind that several other factors in the uremic condition such as inflammation, uremic toxins, calciprotein particles (CPPs), loss of local and circulating inhibitors, and renal injury factors all have adverse effects on the vasculature.^(^ ^17^, ^18^, ^19^, ^20^, ^21^ ^)^ The presence of vascular calcification already occurs at mild to moderate kidney disease, and CKD patients suffer from both intima and medial vascular calcification.^(^ ^22^ ^)^ The latter is predominant in CKD.^(^ ^23^ ^)^

Calcification of the vessel's medial layer is a highly cell‐regulated process, characterized by the phenotypic conversion of the contractile vascular smooth muscle cell (VSMC) into a bone‐like secretory cell, which secretes extracellular proteins in which hydroxyapatite crystals can precipitate.^(^ ^24^ ^)^ Using the RNA‐sequencing technique, our group has studied the dramatic changes in gene profile and demonstrated significant upregulation of signal molecules related to Wnt and transforming growth factor β (TGF‐β) signaling in the calcified aorta from uremic rats.^(^ ^25^ ^)^ Because these factors are found in plasma and at higher levels in CKD,^(^ ^26^, ^27^, ^28^, ^29^, ^30^, ^31^ ^)^ we speculated whether these signal molecules were secreted by the vessel and had endocrine effects on bone metabolism.

To investigate our hypothesis that CKD‐induced vascular calcification directly affects bone metabolism, we transplanted the calcified aorta from uremic rats into normal recipient rats in a previous study.^(^ ^16^ ^)^ Interestingly, we found that the presence of the uremic calcified graft resulted in lower osteoid area and lower mineral density of the trabecular tissue, illustrating a significant impact on bone formation and mineralization.^(^ ^16^ ^)^ Therefore, the aim of the present study was to study our hypothesis of a pathological vascular–bone tissue crosstalk further in elaborated in vitro experiments. Normal aortas and calcified aortas from uremic rats were incubated ex vivo to detect secretion of Wnt inhibitors and activin A. In addition, aorta rings were co‐incubated with the osteoblast‐like cell line UMR‐106 to study their effect on mineralization and signal pathways.

## Materials and Methods

### Aorta tissue

The normal aorta was removed from normal male Wistar rats (8 weeks old) and the uremic calcified aorta was removed from 5/6 nephrectomized Wistar rats (22 weeks old) treated with the active vitamin D analog alfacalcidol 80 ng intraperitoneally (ip) 3 times weekly for 6 weeks, and fed a high 1.4% phosphorus diet (Charles Rivers, Köln, Germany).^(^ ^16^, ^25^ ^)^ For further details, please see the Supplementary Methods.

### Bone cell line

The osteoblast‐like cell line UMR‐106 was purchased from ATCC (CRL‐1661; ATCC, Teddington, UK). Cells were grown in six‐well or 12‐well cell plates (polystyrene, nunclon delta surface; Thermo Fisher Scientific, Waltham, MA, USA) suspended in Dulbecco's modified Eagle medium (DMEM) high glucose (30‐2002; ATCC) supplemented with 10% fetal bovine serum (FBS, heat inactivated, 30‐2025; ATCC) and 1% penicillin/streptomycin (P/S; Gibco, Thermo Fischer Scientific) and incubated at 37°C in a 5% CO_2_ atmosphere. For the mineralization studies, the UMR‐106 cells were grown in 24‐well a Corning Costar transwell system with a pore size of 5.0 μm (3421; Corning Incorporated, Kennebunk, ME, USA). The complete media was added 10mM β‐glycerolphosphate and 50 μg/mL ascorbic acid (G9422‐10G and A4403; Sigma Life Science, St. Louis, MO, USA).^(^ ^32^ ^)^

### Experimental in vitro protocols

1.  Incubation of normal and uremic aorta rings for 24 or 48 hours.
2.  Mineralization study: UMR‐106 cells co‐incubated with normal and uremic calcified aortas for 8 days.
3.  Cell signaling study: UMR‐106 cells co‐incubated with normal and uremic calcified aortas (24 hours). A further study was conducted with or without lithium chloride (LiCl).

### Aorta culture

The aortas from six normal rats and six uremic rats were cultured ex vivo. Under sterile conditions, the whole aorta was gently excised (from arcus aorta to aortic bifurcation) under a stereomicroscope. The vessel was flushed with heparin/saline and placed in cold (5°C) isotonic saline. While suspended in saline, the aorta was gently cut into 1‐mm rings under a stereomicroscope. Then the aorta rings were quickly placed in DMEM high glucose (D5796; Sigma Life Science) supplemented with 10% FBS and 1% P/S. Eight aorta rings were placed per well in a 12‐well cell plate, suspended in 1 mL complete media, and incubated at 37°C in a 5% CO_2_ atmosphere. The culture time was either 24 hours or 48 hours, after which the media was collected and its concentration of sclerostin, dickkopf‐1 (Dkk1), secreted frizzle‐related protein 4 (SFRP4), and activin A were measured using commercial ELISAs, previously evaluated in our laboratory.^(^ ^16^, ^33^ ^)^ Viability of the aorta rings was confirmed by Trypan blue staining.

### The mineralization study

UMR‐106 cells were seeded at a density of 50,000/plate (lower chamber, 24‐well, Transwell). After 1 day of incubation, mineralization media was added followed by insertion of three normal aorta rings or three uremic calcified aorta rings into each insert of eight wells, respectively. Eight wells were incubated without aorta tissue. The media and aorta rings were changed every 2 days. After 8 days of co‐incubation, the UMR‐106 cells were fixed and stained with Alizarin red for assessment of calcium‐containing crystal formation. The experiment was repeated three times with a total of 12 normal aortas and 12 uremic calcified aortas used.

### The cell signaling study

UMR‐106 cells were seeded at a density of 1M in a six‐well plate (2 mL media). At 80% confluence, five aorta rings from normal or uremic rats were placed at the circumference of each well. After 24 hours, the aorta was removed and the UMR‐106 cells were analyzed for genes related to mineralization and Wnt pathway. The experiment was repeated eight times with a total of eight normal and eight uremic calcified aortas used. In addition, a further study was conducted where UMR‐106 cells were incubated with normal and uremic calcified aortas (*n* = 5, 12‐well plate, four aorta rings/well, 1 mL media) with or without 0.05mM LiCl. The dose of 0.05mM LiCl was confirmed to influence Wnt pathway by significantly increasing β‐catenin protein in UMR‐106 cells (Supplemental Methods).

### Biochemistry analyses

The cell culture media was measured for its concentration of sclerostin (R&D Systems, Minneapolis, MN, USA), Dkk1 (NBP2‐61303; Novus Biologicals, Centennial, CO, USA), SFRP4 (LS‐F8488; LSBio, Seattle, WA, USA), and activin A (DAC00B; R&D systems).^(^ ^16^, ^33^ ^)^ The media sample was measured non‐diluted and in dilutions of 1:2, 1:10, and 1:20. The lowest dilution is presented in Results. The plasma from the normal and uremic rats were measured for creatinine, urea, and phosphate using a Vitros 250 analyzer (Ortho‐Clinical Diagnostics, Raritan, NJ, USA). Ionized calcium, electrolytes, hemoglobin, and bicarbonate were measured at actual pH by an ABL 900 (Radiometer, Copenhagen, Denmark). The media's calcium concentration was measured by an ABL 900.

### Aorta calcium

Aortic calcium content was quantified by the o‐cresolphthalein method and normalized to dry weight.^(^ ^34^ ^)^ To illustrate the calcification of the aorta, the proximal thoracic aorta from normal and uremic rats was stained with von Kossa (Supplemental Methods).^(^ ^25^, ^35^ ^)^

### Mineralization analysis by Alizarin red stain

UMR‐106 cells were fixed in 10% formalin for 30 minutes. After rinsing with distilled water (dH_2_O), the cells were stained with 2% Alizarin red (pH 4.2) for 10 minutes, followed by thorough rinsing with dH_2_O.^(^ ^36^ ^)^ Images were acquired using a Zeiss Axio Observer 7 inverted microscope using an EC Plan‐Neofluar 5×/0.16 objective fitted with an Axiocam 105 color camera (Carl Zeiss AG, Oberkochen, Germany). Number and size of calcium‐containing crystals were counted using ImageJ software (NIH, Bethesda, MD, USA; https://imagej.nih.gov/ij/) (Supplemental Methods). The mean of the total number and area of calcium containing crystals of UMR‐106 were used as reference and set to 1.

### Western blot

Protein analysis was performed using standard methods (Supplemental Methods).^(^ ^37^ ^)^ Primary antibodies: 1:5000 total β‐catenin (detects C‐terminal region of β‐catenin; 610153; BD Biosciences, Franklin Lakes, NJ, USA), 1:1000 active β‐catenin (detects nonphosphorylated β‐catenin at Ser37 and Thr41; 05‐665; Merck Millipore, Burlington, MA, USA), 1:1000 osteopontin (ab8448; Abcam, Cambridge, UK). Park7 (ab18257; Abcam) was used as reference protein. The density of protein bands was quantified in Image J. Uncropped WB images are shown in Supplemental Fig. S2.

### Gene analysis by quantitative RT‐PCR

Gene analysis was performed using standard methods (Supplemental Methods).^(^ ^38^ ^)^ The mRNA levels were normalized to the mean of the stable reference genes: *Arbp* and *Rpl13*, and results are shown as the ratio to the mean expression level of control group (eg, normal aorta or UMR‐106) using the delta‐delta comparative threshold cycle (∆∆Ct) method.^(^ ^39^ ^)^ Primers are listed in Supplemental Table S1.

### Statistics

Normal distributed data are expressed as mean ± SD. Skewed data are shown as median [range]. Data are presented in box‐plots showing median, interquartile range, and all data points in all figures. Statistical significance was tested using two‐sided *t* test for data with normal distribution and the Mann‐Whitney *U* test as nonparametric test. One‐way ANOVA followed by Tukey's multiple‐comparison tests was used to compare means between the three groups. All calculations were performed in Prism 8.0 (GraphPad Software, Inc., La Jolla, CA, USA). Significance level was set at *p* < 0.05.

## Results

### Normal and uremic rats' plasma biochemistry and aorta characteristics

To describe the donors of the normal and uremic calcified aortas, the plasma biochemistry of normal and uremic rats is shown in Table 1 and Supplemental Table S2. The uremic rats suffered from severe CKD with severely increased creatinine levels and urea, slightly lower hemoglobin, and significant electrolyte derangements. The phenotypic shift of the uremic calcified aorta was characterized by a strong downregulation of the VSMC markers α‐smooth muscle actin and elastin (Supplemental Fig. S1). The osteogenic transcription factor runt‐related transcription factor 2 (RUNX2) was significantly upregulated, whereas no statistical difference was found in bone morphogenetic protein 2 (BMP2). A high upregulation of the mineralization inhibitor osteopontin was found in the uremic calcified aorta (Supplementary Fig. S1). The uremic calcified aorta had significant higher calcium content in comparison with the normal aorta (Table 1).

**Table 1:** Plasma Biochemistry and Aorta Calcium Content of Normal and Uremic Rats

Group	Creatinine (μM)	Ca^2+^ (mM)	Phosphate (mM)	Sclerostin (pg/mL)	Dkk1 (pg/mL)	SFRP4 (ng/mL)	Activin A (pg/mL)	Aorta calcium (μg Ca/mg weight)
Normal rat	20 ± 8	1.38 ± 0.05	3.18 ± 0.63	359 ± 38	1117 ± 186	11 ± 3	95 [84–139]	0.37 [0.35–0.44]
Uremic rat	123 ± 60	1.26 ± 0.16	4.23 ± 1.49	430 ± 222	1828 ± 1140	42 ± 22	78 [40–283]	1.04 [0.54–56.32]
*p*	<0.001	0.013	0.051	0.471	0.188	0.003	0.390	<0.001

Plasma parameters of kidney function, mineral balance (*n* = 12/12), the Wnt inhibitors: sclerostin, dickkopf 1 (Dkk1), secreted frizzle‐related protein 4 (SFRP4), and TGF‐β member activin A (*n* = 6 or 9). Data are shown as mean ± SD or median [range]. The aorta calcium content was quantified by the o‐cresolphthalein method and normalized to dry weight (*n* = 8).

### The uremic calcified aorta secretes several signal molecules related to the Wnt and TGF‐β pathways

**Fig. 1:**
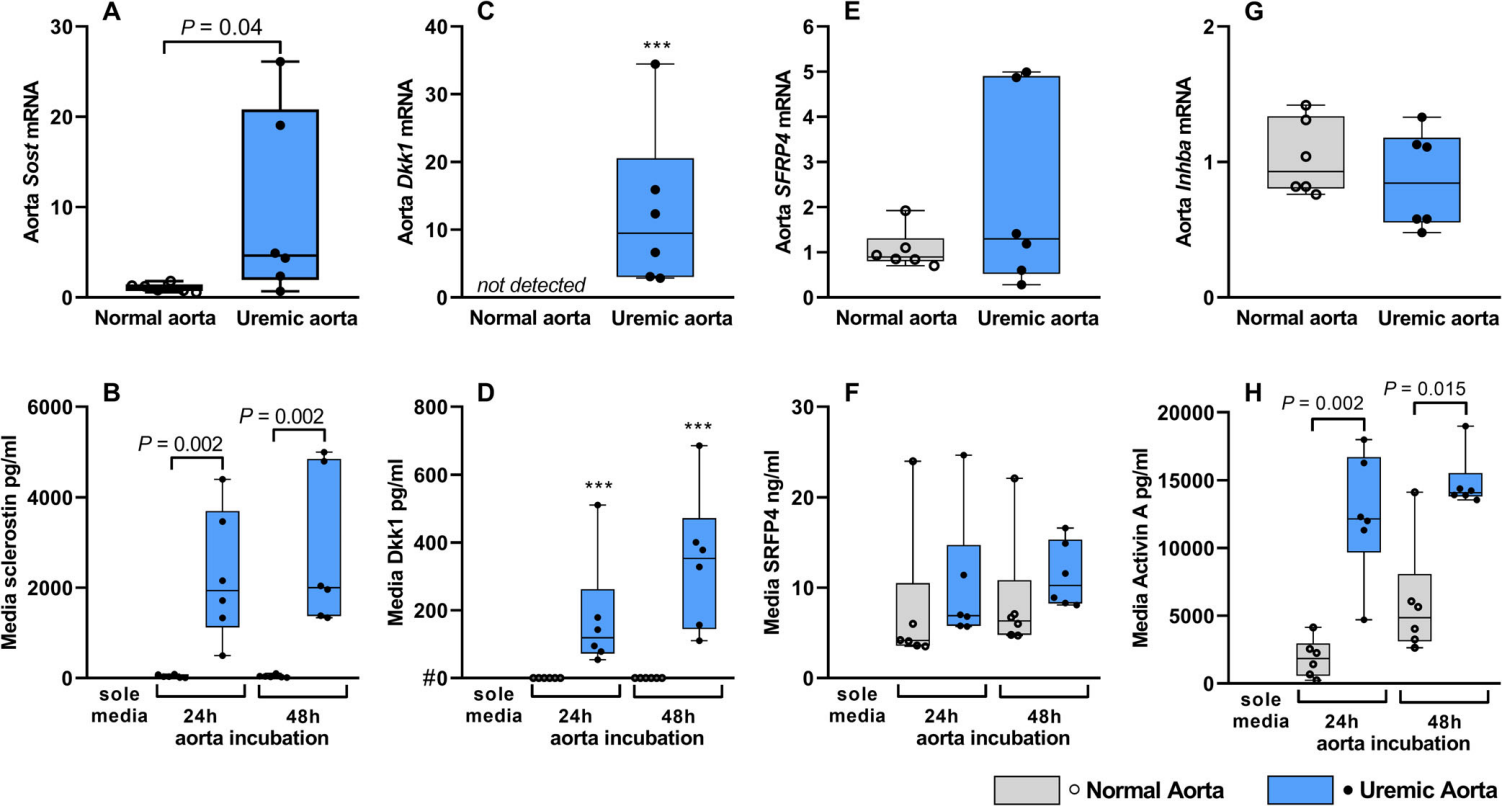
The uremic calcified aorta secretes the Wnt inhibitor sclerostin. (*A*) *Sost* gene, coding for sclerostin, is highly upregulated in the calcified aorta from uremic rats (*p* = 0.04). (*B*) Incubation of 1 mm aorta rings from six normal rats and six uremic rats (eight rings were incubated in 1 mL media/well). Media was collected from one well after 24 hours or 48 hours and sclerostin was measured by ELISA. The calcified aorta from uremic rats secreted high amounts of sclerostin. (*C*,*D*) The Wnt inhibitor dickkopf 1 (Dkk1) was induced in the uremic calcified aorta, which secreted significant amounts of Dkk1 protein into the media (***significant induction and secretion). Dkk1 was not expressed in the normal aorta. (*E*,*F*) Secreted frizzle‐related protein 4 (SFRP4) was expressed in the normal aorta yet varied expression in the uremic calcified aorta. Still, similar secretion of SFRP4 was found. (*G*,*H*) *Inhba* coding for activin A was similar expressed in the normal aorta and uremic calcified aorta. However, the uremic calcified aorta secreted significant higher amounts of activin A into the media. mRNA levels were normalized to the mean of stable housekeeping genes, and results are shown as the ratio to the expression level of normal rats using the ∆∆Ct method. Data are presented in box‐plots showing median, interquartile range and all data points. *n* = 6. #The measurement of dkk1 in the sole media was set to 0 and the other measurement was subtracted.

*Sost* gene coding for sclerostin is primarily expressed by the osteocytes.^(^ ^40^ ^)^ In the uremic calcified aorta the expression of *Sost* was significantly increased (*Sost* mRNA levels median 4.64 [0.68–26.11] versus normal aorta 1 [0.55–1.82], *p* < 0.04, Fig. 1*A*). To assess whether sclerostin was secreted by the calcified aorta from uremic rats, aorta rings were incubated ex vivo for 24 or 48 hours. Sclerostin could not be detected in the sole media (without aorta tissue) at the two time points. Minor secretion of sclerostin was detected by the normal aorta, 31 [7–88] pg/mL 24 hours and 30 [13–104] pg/mL 48 hours. The uremic calcified aorta rings secreted large amounts of sclerostin into the media, more specifically 1936 [495–4400] pg/mL 24 hours and 2001 [1336–5000] pg/mL 48 hours, *p* < 0.002, compared to normal aorta, respectively (Fig. 1*B*).

To further study if the vasculature secreted other signal molecules, the media from the incubated normal and uremic calcified aorta rings was also examined for other factors induced in CKD. Expression of the Wnt inhibitor Dkk1 was induced in the uremic calcified aorta, whereas no transcript was found in the normal aorta (Fig. 1*C*). Moreover, Dkk1 was secreted by the uremic calcified aorta (119 [78–551] pg/mL 24 hours, 353 [110–686] pg/mL 48 hours, Fig. 1*D*). The gene coding for SFRP4 was expressed at similar levels in normal and uremic calcified aortas, and both vessels secreted similar amounts of SFRP4 (Fig. 1*E*,*F*). The uremic calcified aorta secreted significant higher amounts of amounts of activin A (12,158 [4712–18,000] pg/mL 24 hours, 14,084 [13,552‐19,000] pg/mL 48 hours versus 1,838 [250–4146] pg/mL 24 hours, 4850 [2,643‐14,129] pg/mL 48 hours; *p* = 0.002 and *p* = 0.015; respectively, Fig. 1*H*), despite the similar expression of *Inhba*, coding for activin A, found in normal and uremic calcified aortas (Fig. 1*G*). Taken together, the uremic calcified aorta secretes several signal molecules, which potentially can affect bone metabolism.

### The presence of uremic vascular calcification inhibits bone cell mineralization

**Fig. 2:**
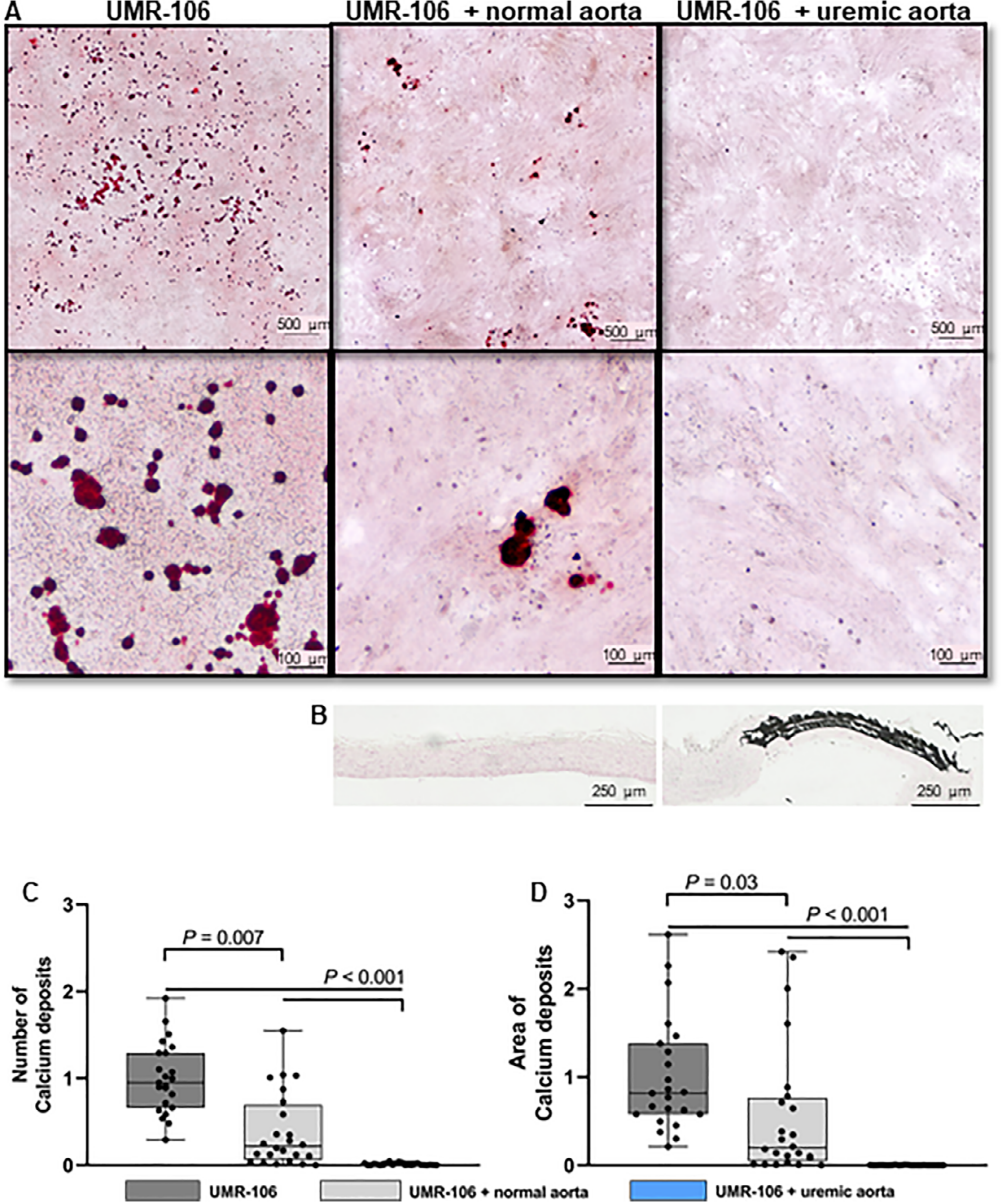
Strong inhibitory effect on bone cell mineralization inflicted by the uremic calcified aorta. (*A*) Representative histological images of the fixed cells stained with Alizarin red in the three groups. The upper images show a large portion of the well. The lower images are enlarged to visualize the calcium containing crystals. (*B*) Histological images of normal and uremic calcified aorta, the latter showed calcium‐phosphate crystals deposition in the media layer (von Kossa stain). (*C*) The number of calcium containing crystals are shown as the ratio to the mean of UMR‐106 (set to 1). Significant fewer mineralization units in UMR‐106 cells incubated with the normal aorta. Strikingly, no mineralization could be detected, when the bone cells were incubated with the uremic calcified aorta. (*D*) The area of mineralization is expressed as the sum of calcium depositions' area and shown as the ratio to the mean of UMR‐106 (set to 1). Less mineralization in UMR‐106 + normal aorta. No mineralization in UMR‐106 cells incubated with the uremic calcified aorta. Data are presented in box‐plots showing median, interquartile range and all data points, where each dot represents the quantification of calcium deposition in an individual well. The experiment was repeated three times with a total *n* of 24 in each group.

To examine whether the calcified aorta from uremic rats had a direct effect on bone cell mineralization, the aorta was co‐incubated with the osteoblast‐like cell line UMR‐106 cells in the mineralization media supplemented with 10mM β‐glycerolphosphate. The media's calcium concentration was measured daily and found similar among groups with an overall mean of 1.23 ± 0.06mM (Supplemental Table S3). The UMR‐106 cells formed calcium containing crystals in the mineralization media (Fig. 2*A*–*D*). When the UMR‐106 cells were co‐incubated with normal aorta rings, they had less calcium depositions (0.22 [0–1.55] versus 1 [0.29–1.92], *p* = 0.007) and a smaller mineralized area (0.20 [0–2.42] versus 1 [0.21–2.62], *p* = 0.03). Nonetheless, a striking effect of the vascular calcification on bone cells was found, as no mineralization could be detected in the UMR‐106 cells co‐incubated with the uremic calcified aorta (Fig. 2*A*–*D*). The normal and uremic aorta tissue absorbed some calcium during incubation, on average 0.82 [0.60–1.26] μg and 0.43 [0.27–28.24] μg Ca/mg dry weight, respectively (~0.02 and 0.01 mmol/mg, after 2 days of co‐incubation). In summary, the uremic calcified aorta strongly inhibited the bone cell's ability to form calcium‐containing crystals.

### The presence of vascular calcification upregulates mineralization inhibitors in bone cells

**Fig. 3:**
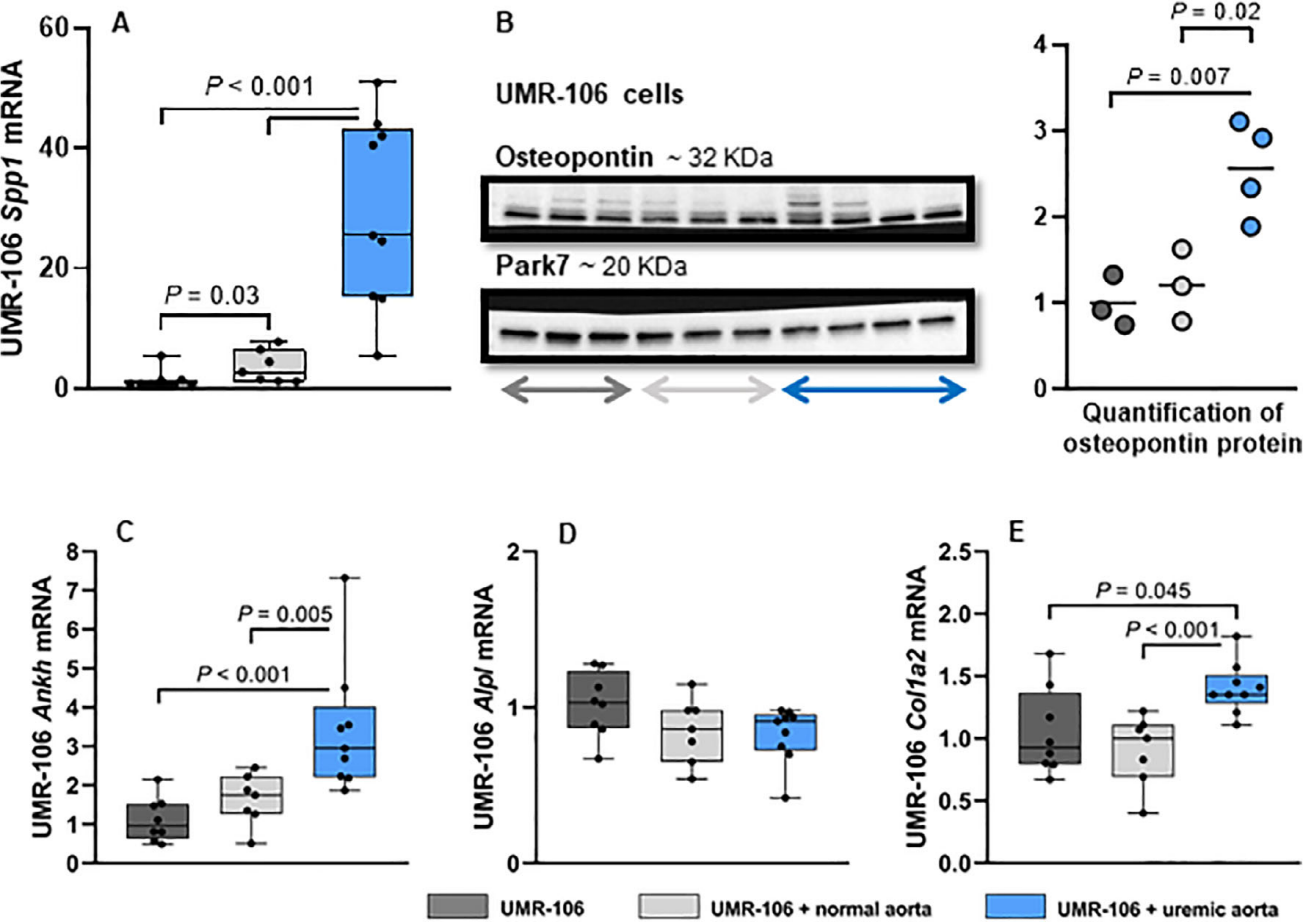
Vascular calcification stimulates expression of mineralization inhibitors in bone cells. The aorta was excised from normal and uremic rats and co‐incubated with UMR‐106 cells for 24 hours. (*A*,*B*) Osteopontin gene (*Spp1*) and protein was upregulated in UMR‐106 cells by the uremic calcified aorta. (*C*) Likewise, another mineralization inhibitor progressive ankylosis protein homolog (ANKH) was also induced by co‐incubation with uremic calcified aorta rings. (*D*) No difference in the expression of alkaline phosphatase (*Alpl*) was found between the three groups. (*E*) Slightly higher expression of collagen I type a2 (*Col1a2*) in UMR‐106 + uremic calcified aorta rings. WB quantified in ImageJ (*n* = 3/4). mRNA levels were normalized to the mean of stable housekeeping genes, and results are shown as the ratio to the expression level of UMR‐106 using the ∆∆Ct method. Data are presented in box‐plots showing median, interquartile range and all data points. *n* = 7/8/9.

The uremic calcified aorta significantly upregulated the mineralization inhibitor osteopontin in UMR‐106 cells after 24 hours of co‐incubation. The gene *Spp1* coding for osteopontin was highly increased (UMR‐106 + uremic aorta 25.50 [5.53–51.00] versus UMR‐106 + normal aorta 2.78 [1.20–7.85] and UMR‐106 1 [0.39–5.49], *p* < 0.001, Fig. 3*A*). The increased osteopontin expression was also confirmed at the protein level by Western blot (Fig. 3*B*). Another mineralization inhibitor progressive ankylosis protein homolog (ANKH) was also significantly upregulated in UMR‐106 cells by the uremic calcified aorta (UMR‐106 + uremic aorta 2.95 [1.87–7.32] versus UMR‐106 + normal aorta 1.75 [0.51–2.46] and UMR‐106 1 [0.49–2.15], *p* = 0.005 and *p* < 0.001, respectively, Fig. 3*C*). No difference was found in the expression level of alkaline phosphatase (Fig. 3*D*). Collagen I was slightly increased in UMR‐106 co‐incubated with the uremic calcified aorta (Fig. 3*E*). So, the bone cells upregulate mineralization inhibitors in the presence of the uremic calcified aorta.

### Involvement of the canonical Wnt/β‐catenin signaling in the vasculature to bone signaling

**Fig. 4:**
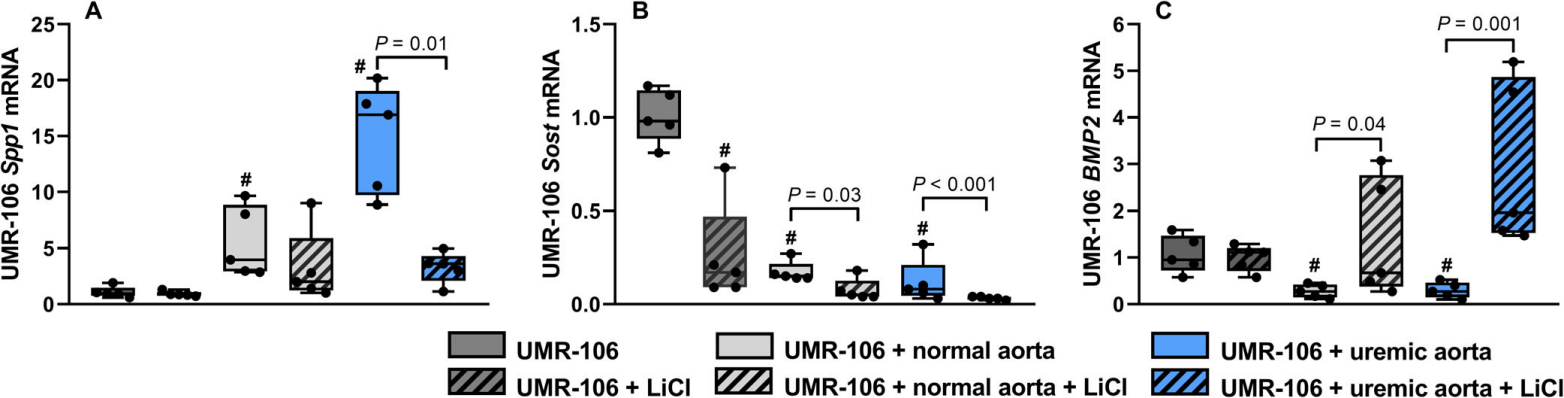
Signals from the vasculature inhibits canonical Wnt/β‐catenin signaling in UMR‐106 cells. The aorta was excised from normal and uremic rats and co‐incubated with UMR‐106 cells for 24 hours with or without 0.05mM lithium chloride (LiCl). (*A*) LiCl stabilizes β‐catenin protein and thereby stimulates its signaling. Administration of LiCl blocked the strong upregulation of osteopontin by the uremic calcified aorta (*B*) UMR‐106 cells expression of *Sost*, coding for sclerostin, was downregulated by LiCl. Co‐incubation with aorta rings had similar effect. (*C*) The expression of bone morphogenetic protein 2 (BMP2) was downregulated by the aorta tissue, yet the effect was abolished by LiCl. mRNA levels were normalized to the mean of stable housekeeping genes, and results are shown as the ratio to the expression level of UMR‐106 using the ∆∆Ct method. Data are presented in box‐plots showing median, interquartile range and all data points. # compared to UMR‐106 *p* < 0.01. *n* = 5.

The uremic calcified aorta secreted both sclerostin and Dkk1. These factors inhibit canonical Wnt/β‐catenin signaling by enhancing degradation of β‐catenin protein. To counteract this possible effect, we added LiCl to the media because it stabilizes β‐catenin protein in UMR‐106 cells. Stabilization of β‐catenin by LiCl hindered the strong induction of osteopontin by the uremic calcified aorta (3.61 [1.14–4.96] versus 16.91 [8.88–20.18], *p* = 0.01, Fig. 4*A*). Furthermore, the effect on expression of *Sost* coding for sclerostin was examined. LiCl significantly downregulated *Sost* expression in UMR‐106 cells. Co‐incubation with aorta rings resulted in similar downregulation and the combined effect was synergistic (Fig. 4*B*). Wnt pathway is interconnected with several other pathways in bone; eg, the anabolic BMP2. Incubation with both normal and uremic calcified aorta resulted in significant downregulation of BMP2 in UMR‐106 cells (0.27 [0.11–0.45] and 0.27 [0.10–0.52] versus 1 [0.58–1.59], *p* < 0.01). This inhibitory effect was also abolished by LiCl administration (Fig. 4*C*). LiCl had no effect on the upregulation of *ANKH* and *Col1a2* by the uremic calcified aorta (Supplemental Fig. S3). In summary, Wnt/β‐catenin signaling is active in UMR‐106 cells, where it regulates the expression of the Wnt inhibitor sclerostin. Some of the signals from the uremic calcified aorta are coupled to the canonical Wnt/β‐catenin pathway in the bone cells.

### Effect of the uremic calcified aorta on β‐catenin and classical Wnt target genes in UMR‐106 cells

**Fig. 5:**
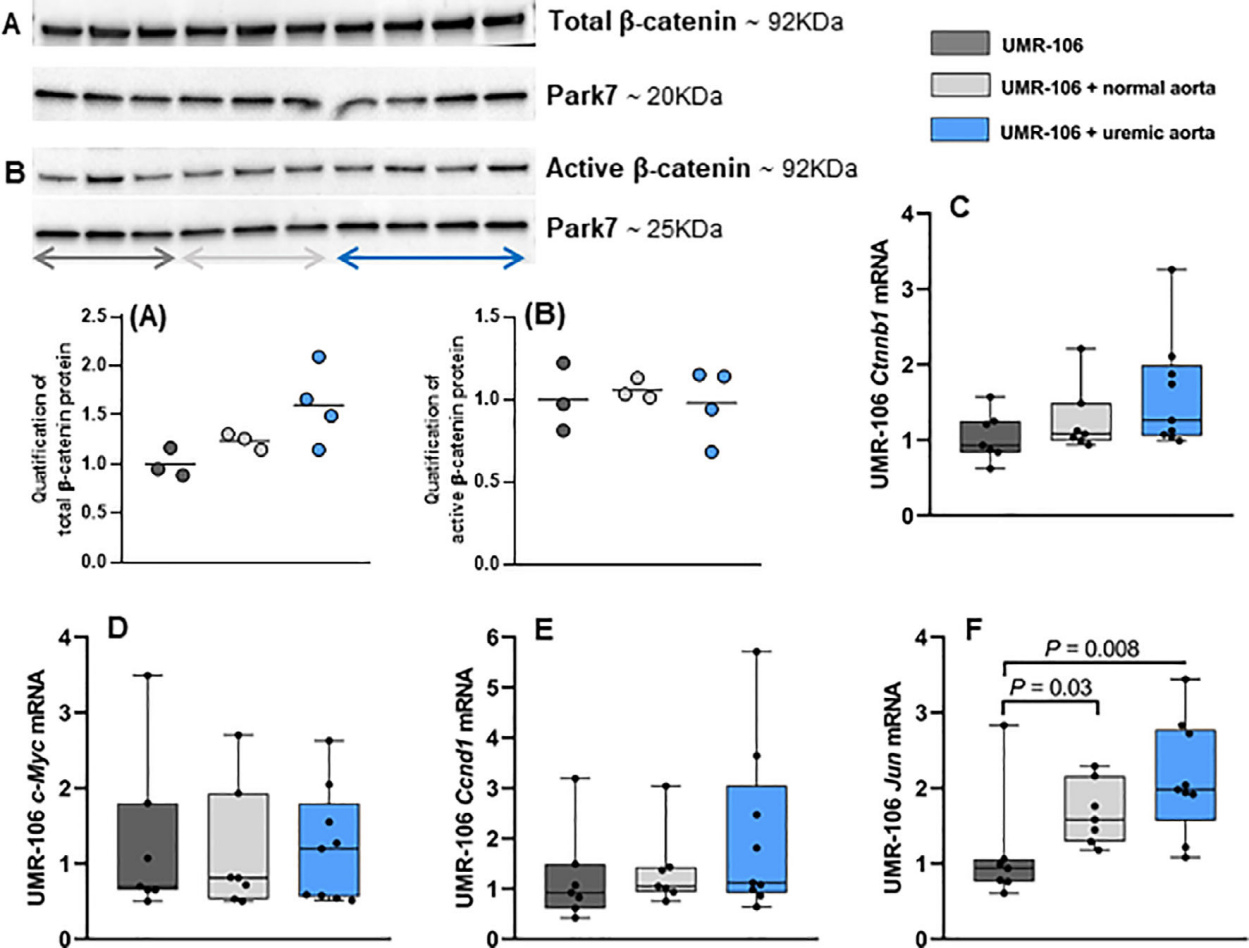
Effect of vascular calcification on Wnt signaling in bone cells. The aorta was excised from normal and uremic rats and co‐incubated with UMR‐106 cells for 24 hours. (*A*,*B*) No difference in total and active β‐catenin protein between the three groups, analyzed by Western blot. (*C*) The expression of the gene *Ctnnb1* coding for β‐catenin protein was similar between all three groups. (*D*–*F*) The expression levels of Wnt target genes *c‐Myc* and *Ccnd1* were similar among the three groups, whereas *Jun* was significantly upregulated by incubation with aorta tissue with no difference between normal and uremic aorta. WB quantified in ImageJ (*n* = 3/4). mRNA levels were normalized to the mean of stable housekeeping genes, and results are shown as the ratio to the expression level of UMR‐106 cells using the ∆∆Ct method. Data are presented in box‐plots showing median, interquartile range and all data points. *n* = 7/8.

**Fig. 6:**
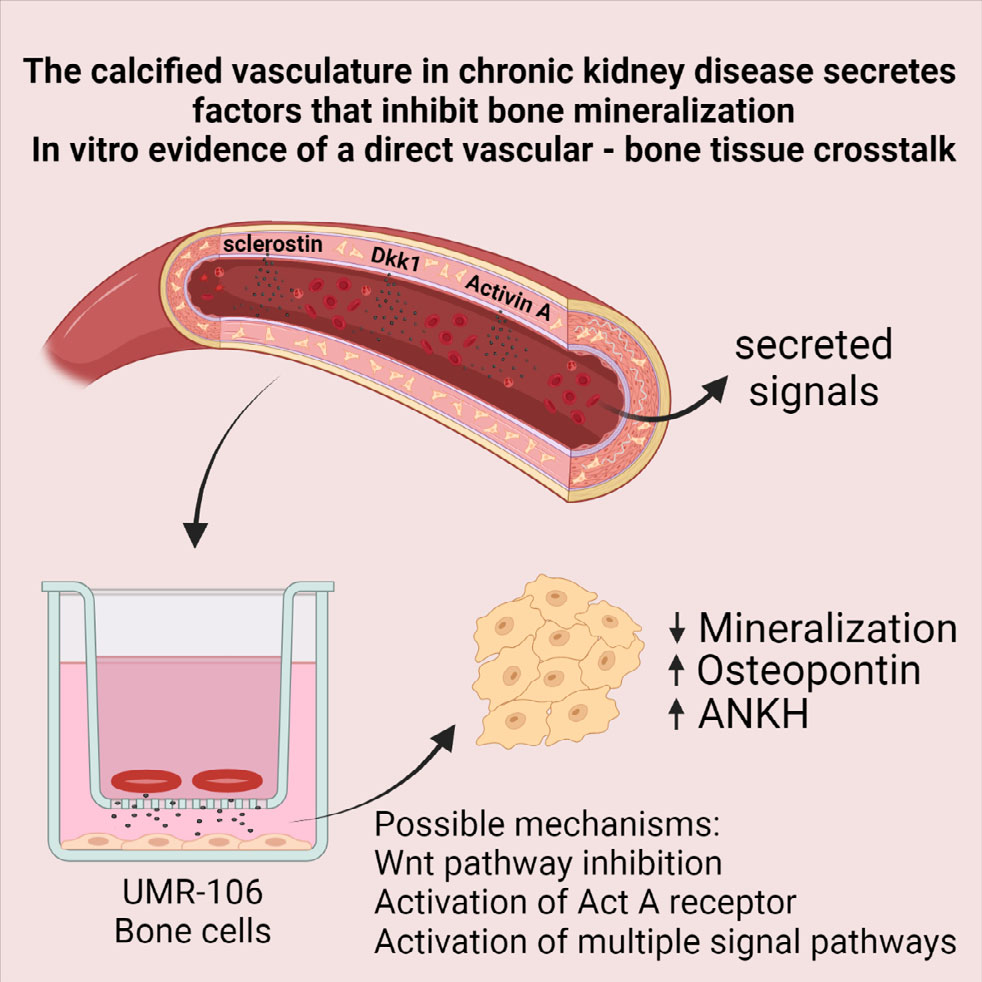
Summary figure. The calcified vasculature in CKD secretes factors that inhibit bone mineralization. The present in vitro study showed that the calcified aorta from CKD rats secreted large amounts of the Wnt inhibitors sclerostin and dickkopf 1 (Dkk1) as well as the TGF‐β ligand activin A. In addition, bone cells exposed for the signaling from the calcified vasculature showed impaired mineralization and upregulation of the mineralization inhibitors osteopontin and progressive ankylosis protein homolog (ANKH). The present study supports the existence of a direct calcified vasculature to bone tissue crosstalk.

Because we found secretion of canonical Wnt/β‐catenin inhibitors by the uremic calcified aorta and were able to block its induction of osteopontin in UMR‐106 cells by stabilizing protein levels of β‐catenin, we examined the effect of the uremic calcified aorta on Wnt signaling in UMR‐106 cells. However, no difference between total and active β‐catenin protein was detected between UMR‐106 cells and UMR‐106 cells co‐incubated with normal and uremic aorta (Fig. 5*A*,*B*). There was no difference in the gene coding for β‐catenin protein between all three groups (*Ctnnb1* mRNA levels: UMR‐106 1 [0.62–1.57], UMR‐106 + normal aorta 1.08 [0.94–2.21], UMR‐106 + uremic aorta 1.26 [0.99–3.26], Fig. 5*C*). The expression levels of Wnt target genes *c‐Myc* and *Ccnd1* were similar among the three groups (*c‐Myc* mRNA levels: UMR‐106 1 [0.50–3.49], UMR‐106 + normal aorta 0.81 [0.50–2.70], UMR‐106 + uremic aorta 1.20 [0.51–2.63] and *Ccnd1* mRNA levels: UMR‐106 1 [0.42–3.19], UMR‐106 + normal aorta 1.05 [0.75–3.04], UMR‐106 + uremic aorta 1.12 [0.64–5.71], Fig. 5*D*,*E*). The transcription factor *Jun*, another Wnt target gene, was significantly upregulated by co‐incubation with aorta tissue, yet no difference was found between normal and uremic aorta (*Jun* mRNA levels: UMR‐106 1 [0.61–2.83] versus UMR‐106 + normal aorta 1.58 [1.18–2.29] and UMR‐106 + uremic aorta 1.98 [1.08–3.44], *p* < 0.03 and *p* < 0.008, respectively, Fig. 5*C*). Adding LiCl to the media had no effect on *Jun* upregulation (Supplementary Fig. S3). Taken together, these observations indicate a constitutive expression of β‐catenin and Wnt target genes in UMR‐106 cells (Fig. 6).

## Discussion

Calcification of the vasculature causes a dramatic shift in the gene expression profile and induction of signal molecules of the Wnt and TGF‐β pathways.^(^ ^25^ ^)^ In a recent study from our group, we showed that vascular calcification impaired bone metabolism.^(^ ^16^ ^)^ Our hypothesis of a direct vasculature to bone–tissue crosstalk was studied in the present in vitro investigation, where it was demonstrated that the Wnt inhibitors sclerostin, Dkk1, SFRP4, and the TGF‐β member activin A were secreted by the calcified aorta from uremic rats. All these secreted signal molecules may affect bone metabolism. In addition, co‐cultures with the osteoblast‐like cell line UMR‐106 showed a powerful effect of the uremic calcified aorta on bone cell mineralization because it abolished formation of calcium‐containing crystals. The mineralization inhibitor osteopontin was highly upregulated in UMR‐106 cells co‐incubated with the uremic calcified aorta; this may represent one of the mechanisms behind the strong antimineralization effect (Fig. 6). The induction of osteopontin was blocked by LiCl, indicating the secreted factors from the uremic calcified aorta inhibit the canonical Wnt/β‐catenin. At the same time, other effects of the aorta on UMR‐106 gene transcription could not be blocked by LiCl, indicating a complex impact on several pathways. This is also in accordance with the findings that several signal molecules were secreted by the uremic calcified aorta.

The complex Wnt pathway consists of multiple ligands, several downstream signaling pathways (canonical β‐catenin and noncanonical), interaction with other pathways such as TGF‐β, BMPs, and several antagonists. In most tissues Wnt signaling is primarily active during embryogenesis but is reactivated in tissue injury and fibrosis.^(^ ^41^ ^)^ However, in bone Wnt signaling continues to be an important anabolic pathway.^(^ ^42^ ^)^ Osteocytes secrete sclerostin, which is an essential factor in balancing bone formation and resorption.^(^ ^40^, ^43^, ^44^ ^)^ In the present study we showed upregulation of *Sost* gene in the calcified aorta from uremic rats, which also secreted sclerostin in high amounts, like our previous results.^(^ ^16^ ^)^ We have previously shown that our model of CKD‐induced vascular calcification was accompanied by a large increase in plasma sclerostin in the uremic rats, which was not reflected in their bone's expression of sclerostin, indicating extraskeletal production.^(^ ^16^ ^)^ Similarly, De Maré and colleagues^(^ ^45^ ^)^ found a gradual increase in plasma sclerostin as the calcification of vasculature progressed without increased bone sclerostin expression in another model of vascular calcification using warfarin treatment. In humans, expression of Sost/sclerostin has been demonstrated in vascular pathology where it co‐localizes with calcification.^(^ ^46^, ^47^ ^)^ Also, plasma sclerostin has been positively linked to parameters of vascular calcification.^(^ ^46^, ^48^, ^49^, ^50^, ^51^ ^)^ Plasma sclerostin increases early in CKD and rises progressively as kidney function decreases,^(^ ^26^, ^29^, ^46^, ^52^ ^)^ although the failing kidneys actually increase the fractional sclerostin excretion.^(^ ^53^ ^)^ Combing the observational and experimental data, extraskeletal production of sclerostin in the calcified vessels may very likely contribute to the increased plasma levels of sclerostin in CKD.

The presence of a uremic calcified aorta strongly inhibited the UMR‐106 cells' ability to mineralize in vitro. These results are in line with our previous findings in vivo, where normal rats transplanted with a uremic calcified aorta developed lower BMD.^(^ ^16^ ^)^ These rats also had a significant upregulation of bone expression of the mineralization inhibitors osteopontin and ANKH,^(^ ^16^ ^)^ similar to the present findings in UMR‐106 cells. Osteopontin is a negative charged bone matrix protein, which, among its multiple functions, has the ability to bind calcium with a high affinity and has been shown to inhibit crystal growth.^(^ ^54^, ^55^ ^)^ In cohort studies, higher plasma levels of osteopontin has been linked to lower BMD.^(^ ^56^, ^57^ ^)^ ANKH is a transmembrane protein that transports inorganic pyrophosphate (PPi), which is a strong inhibitor of hydroxyapatite mineralization.^(^ ^58^, ^59^ ^)^ Adding recombinant sclerostin and activin A to bone cells inhibits their mineralization.^(^ ^60^, ^61^ ^)^ Accordingly, the impairment in UMR‐106 cells' mineralization could be explained by the secreted sclerostin and/or activin A from the calcified aorta. Still, there may be other factors secreted by the aorta not examined in the present investigation. For example, inflammatory cytokines are known to have a negative impact on bone metabolism.^(^ ^62^ ^)^ Interestingly, we found the same upregulation of osteopontin in the uremic calcified aorta as well as in bone tissue in vivo and bone cells in vitro exposed for signaling from the calcified artery.^(^ ^16^ ^)^ These findings underline the close link between the pathological processes in vessels and bone, and they may provide a mechanistic explanation to the common finding of lower BMD and presence of vascular calcification in human cohorts.

Sclerostin and Dkk1 bind the co‐receptor LRP5/6 of the Wnt receptor complex (frizzle receptor and LRP5/6) and so antagonize the Wnt/β‐catenin canonical pathway.^(^ ^63^, ^64^ ^)^ In the absence of Wnt activation, β‐catenin's degradation complex consisting of adenomatous polyposis coli (APC), axin and glycogen synthase kinase 3 (GSK3) phosphorylate β‐catenin, which results in rapid degradation.^(^ ^42^ ^)^ As both sclerostin and Dkk1 were secreted by the uremic calcified aorta, lower β‐catenin protein levels would be expected in the co‐incubated UMR‐106 cells. However, the same levels of total and active β‐catenin were found in all three groups. Because the presence of aorta tissue strongly downregulated *Sost* mRNA in UMR‐106 cells, it may have affected the balance between endogenous and exogenous sclerostin and their effects on β‐catenin levels. Classical canonical Wnt target genes c‐Myc and Cyclin D1 were also not significantly altered in all three groups. These findings are in contrast to the ones found in our animal model of aorta transplantation, where the recipients of the uremic calcified graft had increased *Sost* expression and downregulation of Wnt target genes.^(^ ^16^ ^)^ Although the present in vitro model is suitable to study direct signaling from the uremic calcified aorta, it is limited to represent the physiological bone formation in vivo, which comprise of a complex interplay between several cell types and multiple cell signaling. Moreover, the UMR‐106 cell line is a clonal derivative of a rat osteosarcoma and so the cells may have altered Wnt signaling in comparison to the osteoblast, because disturbed Wnt signaling is commonly found in cancer.^(^ ^65^ ^)^ The precise downstream signaling pathways of Wnt/β‐catenin in bone are largely unknown.^(^ ^66^ ^)^

Stabilizing β‐catenin signaling by LiCl blocked the strong induction of osteopontin, indicating a key role of Wnt/β‐catenin pathway in the vasculature to bone cell signaling. LiCl binds GSK of the β‐catenin's degradation complex thus increases β‐catenin protein and signaling.^(^ ^67^, ^68^ ^)^ LiCl also alleviated the normal and uremic calcified aortas' effect on *BMP2* and potentiated the downregulation of *Sost*. Whether we failed to detect the possible changes in β‐catenin protein by Western blot analysis, or our findings are a result of a complex interplay between several signals, where increased β‐catenin levels counteract the effects of the other factors, is not clear. Still, other experimental studies have shown that disturbed Wnt/β‐catenin signaling may be involved in the development of renal osteodystrophy. Sabbagh and colleagues^(^ ^69^ ^)^ used a model of progressive renal failure (*jck* mouse) and found an early increase in bone sclerostin before alterations in the classical CKD‐MBD parameters. Fang and colleagues^(^ ^28^ ^)^ treated diabetic mice with stage 2 CKD with anti‐Dkk1 antibodies and found positive effects on bone volume. In a study by Moe and colleagues^(^ ^70^ ^)^ CKD rats with low or high bone turnover were treated with anti‐sclerostin antibodies. Neutralization of sclerostin only improved bone volume in the low bone turnover condition. The role of circulating sclerostin on bone metabolism is not clarified, partly due to the rather contradictory finding of a positive association between plasma sclerostin and bone BMD in both CKD and population cohorts.^(^ ^48^, ^71^, ^72^, ^73^, ^74^, ^75^, ^76^, ^77^ ^)^ Still, the studies all point toward an involvement of disturbed Wnt pathway in bone disorders in CKD. Future research is needed to elucidate the detailed pathophysiological mechanisms of the different subtypes of renal osteodystrophy, and to illustrate how the calcified vasculature affects bone metabolism along the course of declining kidney function.

Even the presence of a normal aorta reduced calcium containing crystal formation in UMR‐106 cells and affected the expression of *Jun*, *Sost*, and *BMP2*. These results suggest that activation of tissue injury and local repair mechanisms may have off target effects. This notion is in line with the new paradigm proposed by Hruska and colleagues^(^ ^21^ ^)^ that kidney injury factors have negative systemic effects in CKD‐MBD. Similar secretion of SFRP4 was found in normal and uremic calcified aortas. SFRP4 is a decoy receptor for Wnt ligand and antagonizes both canonical and noncanonical Wnt signaling.^(^ ^78^ ^)^ Its exact role in bone homeostasis is not yet well understood. Plasma levels of SFRP4 were four times above normal levels in the uremic rats, suggesting a disturbed SFRP4 balance in CKD. Still, conflicting results have been found in patients with CKD.^(^ ^31^, ^79^ ^)^

Recently, activin A has been proposed to play a role in the CKD‐MBD.^(^ ^21^ ^)^ Activin A is widely distributed in bone and seems to be a negative regulator of bone turnover by several mechanisms on formation and resorption.^(^ ^80^ ^)^ The cytokine is induced in kidney disease, and our group recently showed that it is secreted by the injured kidney.^(^ ^26^, ^33^, ^81^ ^)^ In the present study, we found secretion of activin A from the normal aorta (ex vivo) and even higher secretion from the uremic calcified aorta. Increased activin A signaling is found in CKD and targeting the pathway has shown beneficial effects on kidney, vasculature, and bone.^(^ ^27^, ^82^, ^83^, ^84^ ^)^

Classical treatment strategies for vascular calcification have limited effect in CKD patients, which still have a very high cardiovascular mortality.^(^ ^85^, ^86^ ^)^ Therefore, it is of high importance to further study whether the local expression of sclerostin, Dkk1, SFRP4, and activin A has a protective or detrimental role in uremic vasculopathy. The use of sclerostin antibodies in the treatment of osteoporosis has raised concerns regarding cardiovascular side‐effects.^(^ ^87^, ^88^, ^89^ ^)^ In a phase II trial of sotatercept (activin receptor type IIA fusion protein trap) effect on hemoglobin in dialysis patients, a positive effect on aorta calcification and BMD was reported.^(^ ^84^ ^)^

Although the present in vitro study enables the studying of direct signals from the uremic calcified vasculature to bone cells, it is limited to elucidate the full role of uremic vascular disease in the complex syndrome of CKD‐MBD. Adding 0.05mM LiCl to the 8‐day mineralization study affected cell viability and so its effect on the calcification of UMR‐106 cells was not possible to clarify. In addition, the study is limited to differentiate between long‐term uremia versus severe vascular calcification as well as a potential effect of age. Another limitation is the use of the rat model, as the rat is known to not easily develop vascular calcifications. The established model of CKD‐induced vascular calcification in the rat therefore uses high doses of alfacalcidol and dietary phosphate.^(^ ^16^, ^25^, ^34^, ^35^, ^90^ ^)^ In this model of CKD‐induced vascular calcification, hormonal levels are responding to not only uremia but also to the treatment with active vitamin D analog, and so plasma level of PTH is suppressed and FGF23 is highly increased as previously shown in the same model.^(^ ^16^, ^25^, ^34^, ^35^ ^)^

In conclusion, the vascular calcification process in CKD induces local expression of the Wnt inhibitors sclerostin, Dkk1, SFRP4, and the TGF‐β family member activin A. These factors are shown to be secreted from the calcified aorta ex vivo. Bone cells co‐incubated with uremic calcified aortas have impaired calcium crystal formation and upregulation of mineralization inhibitors. The present study supports the existence of a pathological vascular–bone tissue crosstalk in CKD‐MBD. Targeting signals from the calcified arteries may represent a new approach for treatment of the severe bone disease in CKD‐MBD.

## Conflict of Interests

All authors declared no competing interests.

### Peer Review

The peer review history for this article is available at https://publons.com/publon/10.1002/jbm4.10610.

## Supporting information


**Supplemental Table S1:** Primers
**Supplemental Table S2**: Biochemistry of normal donor and uremic donor
**Supplemental Table S3**: The media's calcium concentration in the 8 days mineralization study
**Supplemental Fig. S1**: The osteogenic shift in the uremic calcified aorta
**Supplemental Fig. S2**: Uncropped WB images
**Supplemental Fig. S3**: The involvement of canonical Wnt/β‐catenin signaling in the vasculature to bone signaling.
