# Supplementary material for: The Calcified Vasculature in Chronic Kidney Disease Secretes Factors that Inhibit Bone Mineralization
Source: JBMR Plus. 2022 Mar 1;6(4):e10610. doi: 10.1002/jbm4.10610 (PMC9009125; doi:10.1002/jbm4.10610)
Supplement: Supplementary file 1 — Supplemental Table S1: Primers Supplemental Table S2: Biochemistry of normal donor and uremic donor Supplemental Table S3: The media's calcium concentration in the 8 days mineralization study Supplemental Fig. S1: The osteogenic shift in the uremic calcified aorta Supplemental Fig. S2: Uncropped WB images Supplemental Fig. S3: The involvement of canonical Wnt/β‐catenin signaling in the vasculature to bone signaling. [file JBM4-6-e10610-s001.docx]

**Supplemental Methods:**

**Aorta tissue from normal and uremic rats**

The normal aorta was removed from healthy male Wistar rats (8 weeks old, n=31) (Charles Rivers, Köln, Germany). The calcified aorta was removed from uremic Wistar rats (22 weeks old, n=31). All rats were housed at an AAALAC accredited facility with a 12-hour light/day cycle and free access to water and food. The standard diet was provided by Safe (Safe D30, Rosenberg, Germany). The CKD-induced vascular calcification model was approved by the Danish Animal Experiments Inspectorate (license no 2017-15-0201-01214). All animal handling was performed in accordance to the national guidelines for care and use of laboratory animals. Animal data are reported according to the ARRIVE guidelines.^1^

**CKD-induced vascular calcification model in the rat**

Chronic uremia was induced by one-step 5/6 nephrectomy as previously described by our laboratory.^2^ Briefly, rats were anesthetized with hypnorm-midazolam (Dept of Experimental Medicine, University of Copenhagen, Copenhagen, Denmark). In a retroperitoneal approach the right renal artery and vein were ligated and the kidney removed. The poles of the left kidney were removed leaving 1/3^rd^ remnant of left kidney tissue. Rats were given carprofen subcutaneously as pain relief for the following 3 days (Rimadyl, Pfizer, Copenhagen, Denmark). To induce vascular calcification in the 5/6 nephrectomy model, the uremic rats were given a high-phosphate diet starting one week after operation (0.9% calcium, 1.4% phosphate, and 600 IU cholecalciferol, Alt 1320 mod, Altromin, Lage, Germany). Eight weeks after surgery, rats were treated with 80 ng alfacalcidol intraperitoneally 3 times weekly for 6 weeks (Leo Pharmaceutical, Copenhagen, Denmark). At the age of 22 weeks (after 14 weeks of uremia) severe vascular calcification has developed in the 5/6 nephrectomized rats, as previously published by our group.^3^

**Lithium chloride (LiCl) titration in UMR-106 cells**

UMR-106 cells were grown in 6 wells cell plate suspended in DMEM, 10% FBS, 1% P/S Gibco, and incubated at 37°C in a 5% CO_2_ atmosphere. At 80% confluence, increasing doses of LiCl was added to the media that is 0.01 mM LiCl, 0.05 mM LiCl and 0.1 mM LiCl. Cells were harvested after 24 hours. Total and active β-catenin protein were measured by Western Blot.


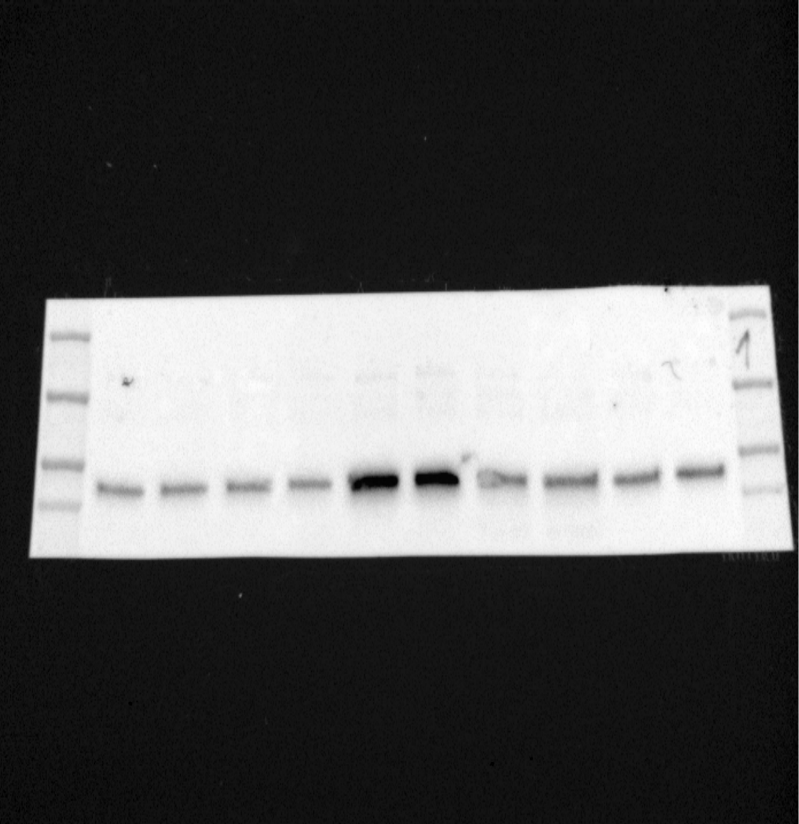

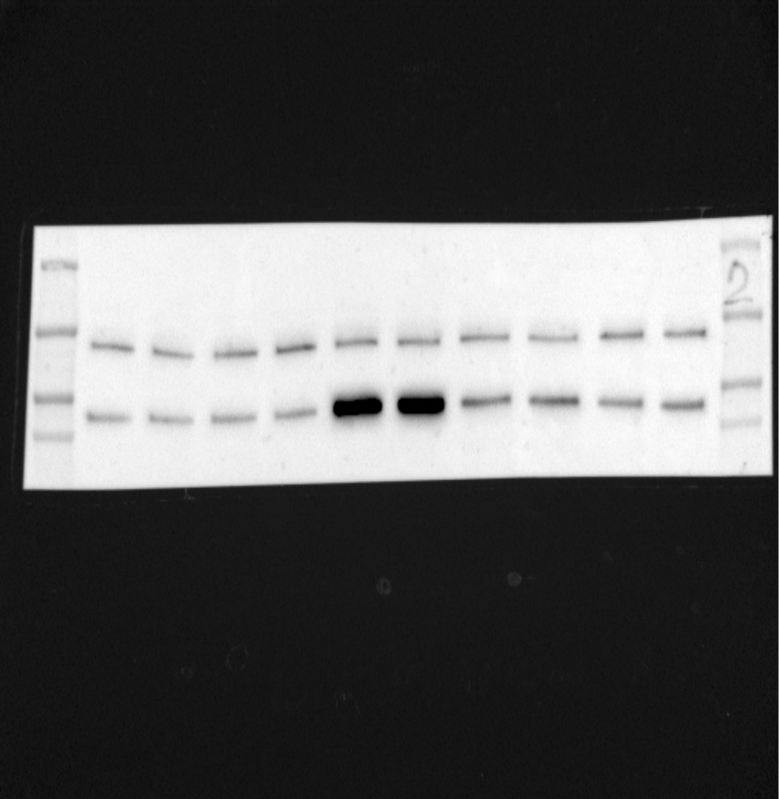

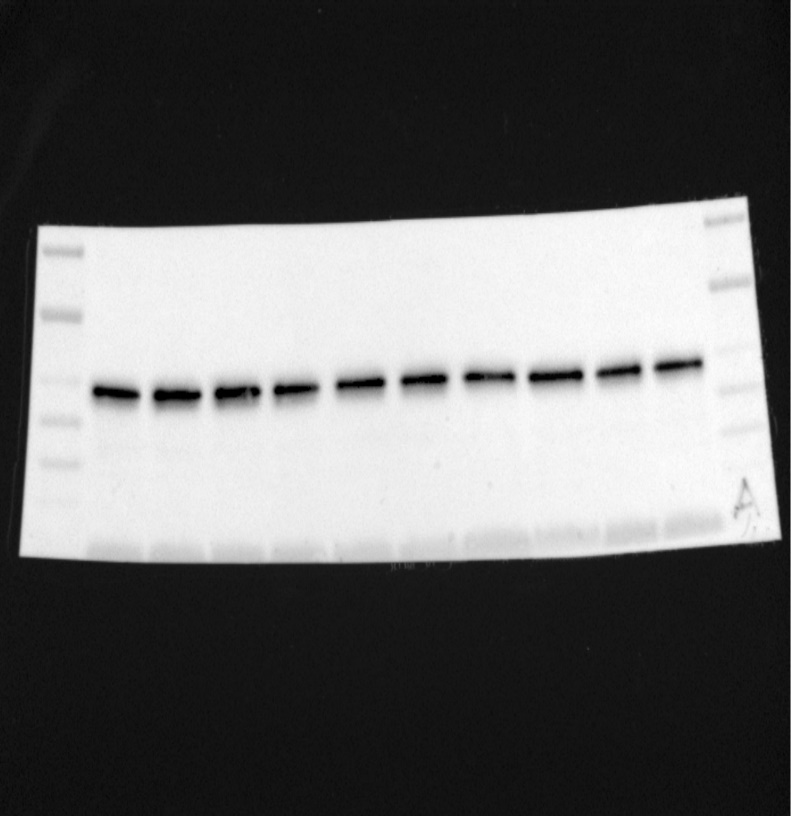

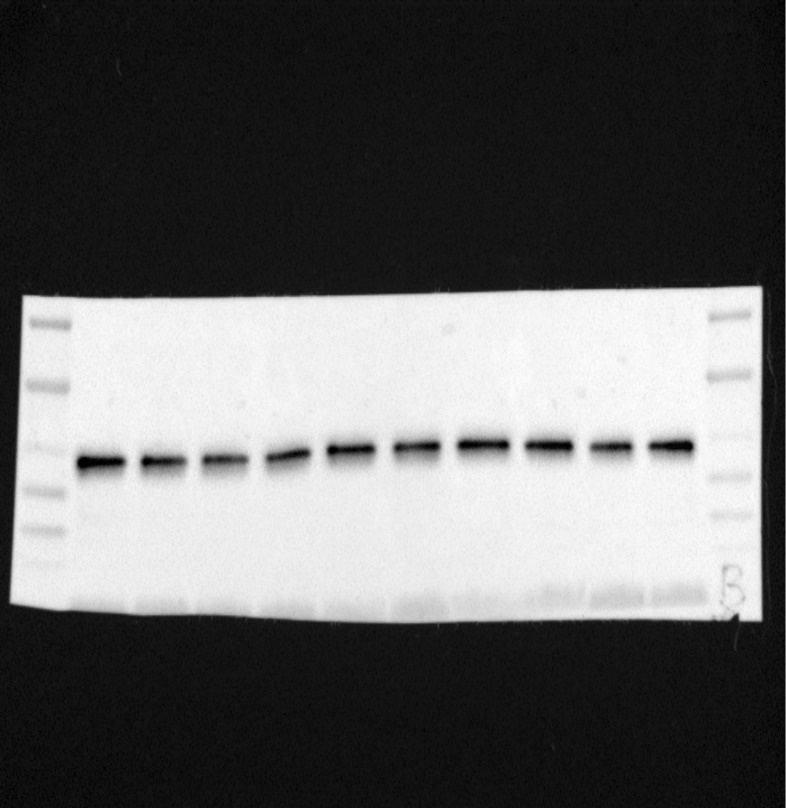


**Reference protein Park7**

**Reference protein Park7**

**Total β-catenin**

**Active β-catenin**

UMR-106

UMR-106

0.01 mM

LiCl

UMR-106

**0.05 mM**

**LiCl**

UMR-106

0.1 mM

LiCl

UMR-106

+ aorta

Normal rat

UMR-106

UMR-106

0.01 mM

LiCl

UMR-106

**0.05 mM**

**LiCl**

UMR-106

0.1 mM

LiCl

UMR-106

+ aorta

Normal rat

Primary antibodies: 1:5000 total β-catenin (610153, BD biosciences), 1:1000 active β-catenin (05-665, Merck Millipore), and 1:1000000 Park7 (ab18257, Abcam). The dose of 0.05 mM LiCl significantly increased total and active β-catenin protein levels.

**Calcium quantification and visualization in the aorta**

Aortic calcium content was determined by the o-cresolphthalein method as previously published.^4^ Briefly, a section of the aorta (n=8/8) was lyophilized for 24 hours to determine dry weight followed by decalcification in 1M HCl for 3 days and so the Ca-content of the supernatant was measured using a commercial calcium colorimetric assay (MAK022-1KT, Sigma Life Science). The proximal thoracic aorta from a normal and uremic rat were fixed in 10% buffered formalin for 24 hours at room temperature. After formalin fixation, tissue sections were dehydrated, embedded into paraffin blocks and cut into 4μm sections. Von Kossa and H&E staining was performed according to standard protocols.^5^ Slides were scanned with Hamamatsu NanoZoomer XR and representative histological images of normal and uremic calcified aorta were made using Hamamatsu NDPview 2.

**Quantification of Alizarin red stain in ImageJ**

The histological images of the fixed UMR-106 cells stained with Alizarin red were opened in ImageJ. To overcome the “edge effect” (eg. non-specific staining of dead cells around the edges of the wells), a quadrant was drawn.

The region of interest (the quadrant) was analyzed. The image was saved as black and white (32bit) and same threshold for Alizarin red stain was used for all images. The number and size of calcium crystals were counted using analyze particle function in ImageJ. The detected stain by ImageJ was visually compared to the original histological image to ensure no artefacts were measured. The mean of the total number and the mean of the total area of UMR-106 calcium crystals were used as reference and set to 1. Therefore, the calcium crystal formation in UMR-106 + normal aorta and UMR-106 + uremic aorta are shown as the ratio to the value of control group UMR-106.

**Western blot**

Protein was extracted from the cell pellets using RIPA buffer and protease inhibitor cocktail (cat no 89900 & 78422, Thermo Fischer Scientific, Waltham, MA, USA). Protein was quantified using Pierce BCA protein assay kit (cat no 23227, Thermo Fischer Scientific) and 30 µg protein was placed in each lane of stain-free precast gel and after electrophoresis transferred to nitrocellulose membrane (456-8085 & 1620112, Bio-Rad, Hercules, CA, USA). Membranes were blocked in milk. Primary antibodies: 1:5000 total β-catenin (detects c-terminal region of β-catenin, 610153, BD biosciences, Franklin Lakes, NJ, USA), 1:1000 active β-catenin (detects dephosphorylated β-catenin at Ser37 & Thr41, 05-665, Merck Millipore, Burlington, MA, USA), 1:1000 osteopontin (ab8448, Abcam, Cambridge, UK). Park7 (ab18257, Abcam) was used as reference protein (1:1000000). Secondary antibodies were anti-mouse 1:1000 and anti-rabbit 1:2000 (p0447 & p0448, Dako, Glostrup, Denmark). Blots were visualized by Amersham ECL Prime detection reagent (RPN2232, GE Healthcare, Freiburg, Germany) using the Chemidoc XRS+ system (Bio-Rad). The density of protein bands was quantified in Image J. Uncropped WB images are shown in Supplementary Figure S2.

**Gene analysis by quantitative RT-PCR**

Total RNA from aorta and UMR-106 cells were extracted using the EZNA RNA isolation kit (Omega Bio-tek, Norcross, GA, USA). Synthesis of cDNA was performed using the Superscript III cDNA kit (Invitrogen, Thermo Fischer Scientific, Waltham, MA, USA). Roche LightCycler 480 (Roche, Basel, Switzerland) with a temperature profile of 94°C for 2 min, 45 cycles of 94°C for 30 sec, 59°C for 45 sec and 72°C for 90 sec and JumpStart (Sigma-Aldrich, St. Louis, MO, USA) were used for quantitative real time PCR. Melting curve analysis was performed to confirm a single PCR product. The mRNA levels were normalized to the mean of the stable reference genes: *Arbp* and *Rpl13.*^3,6^ Results are shown as the ratio to the mean expression level of control group (eg. normal aorta or UMR-106 incubated without aorta tissue) using the ∆∆Ct method.^7^ Primers are listed in Table S1.

1. Kilkenny C, Browne WJ, Cuthill IC, et al. Improving bioscience research reporting: the ARRIVE guidelines for reporting animal research. PLoS Biol. 2010;8:e1000412.

2. Lewin E, Colstrup H, Pless V, et al. A model of reversible uremia employing isogenic kidney transplantation in the rat. Reversibility of secondary hyperparathyroidism. Scand J Urol Nephrol. 1993;27:115-120.

3. Rukov JL, Gravesen E, Mace ML, et al. Effect of chronic uremia on the transcriptional profile of the calcified aorta analysed by RNA-sequencing. Am J Physiol Renal Physiol. 2016;310:F477-91.

4. Gravesen E, Lerche Mace M, Nordholm A, et al. Exogenous BMP7 in aortae of rats with chronic uremia ameliorates expression of profibrotic genes, but does not reverse established vascular calcification. PLoS One 2018;13:e0190820.

5. Gravesen E, Nordholm A, Mace M, et al. Effect of inhibition of CBP-coactivated beta-catenin-mediated Wnt signalling in uremic rats with vascular calcifications. PLoS One 2018;13:e0201936.

6. Mace ML, Gravesen E, Nordholm A, et al. Chronic Kidney Disease-Induced Vascular Calcification Impairs Bone Metabolism. J Bone Miner Res. 2021;36:510-522.

7. Livak KJ, Schmittgen TD. Analysis of relative gene expression data using real-time quantative PCR and the 2(-Delta Delta C(T)) Method. Methods 2001;25:402-408.

**Supplemental Table S1: Primers**

| Gene symbol | Name | Primer sequence |
| --- | --- | --- |
| *Acta2* | α-Smooth muscle actin | F: CGGCGGGCATCCACGAAACC  R: GAGCCGCCGATCCAGACAGAAT |
| *Alpl* | Alkaline phosphatase | F: atgtggactacctattgggtctct  R: cgtggtcaattctgcctccttcca |
| *Ankh* | Progressive ankylosis protein homolog | F: gggcgacgcaaccataagaaagat  R: tgtgggccgaggtgaccgtgttgt |
| *Arbp (Rplp0)* | Ribosomal protein lateral stalk subunit P0 | F: aaagggtcctggctttgtct  R: gcaaatgcagatggatcg |
| *Bmp2* | Bone morphogenetic protein 2 | F: CACAGGGACACACCAACCAT  R: GCCACGATCCAGTCATTCCA |
| *Ctnnb1* | β-catenin | F: tccaggaatgaaggcgtggcaaca  R: cagtccgagatcagcagtctca |
| *Col1a2* | Collagen I, α2 | F: ttggccgaactggagaaatagg  R: gaataccgggagcaccaagaagac |
| *Dkk1* | Dickkopf 1 | F: CACGGTGCCGGGGATGGATA  R: GCCTGGAAGAATTGCTGGTTTGAT |
| *Eln* | Elastin | F: GTGGAGTTGGCCCTGGTGGTGTTA  R: GCAGCCGCCTTAGCAGCAGATTT |
| *Myc* | C-Myc | F: GCTCTCCGTCCTATGTTGCG  R: TCGGAGACCAGTTTGGCAG |
| *Ccnd1* | Cyclin D1 | F: cctctcctgctaccgcacaa  r: cgcaggcttgactccagaag |
| *Spp1* | Osteopontin | F: ccgaggtgatagcttggctt  R: tcggactcctggctcttcat |
| *Rpl13* | Ribosomal protein L13a | F: CCCTCCACCCTATGACAAGA  R: CCTTTTCCTTCCGTTTCTCC |
| *Sfrp4* | Secreted frizzle-related protein 4 | f: GGTCCTTTGATGCTGACTGTAAAC  R: TGGCATGAATAACATAGCTGTAG |
| *Sost* | Sclerostin | F: gcctcctcaggaactagagaac  R: tactcggacacgtctttggtg |

**Supplemental Table S2: Biochemistry of normal donor and uremic donor**

|  | Urea  (mM) | Hemoglobin  (mM) | HCO3-  (mM) | Potassium  (mM) | Sodium  (mM) |
| --- | --- | --- | --- | --- | --- |
| Normal Rat (n=12 ) | 5±0.5 | 8,6±0,4 | 28±2 | 4,2±0,3 | 141±1 |
| Uremic Rat (n=12 ) | 18±10 | 7,8±1,2 | 28±6 | 5,0±0,7 | 144±3 |
| *P*-value | 0.001 | 0.02 | 0.71 | <0.001 | 0.001 |

Plasma biochemistry of normal and uremic rats. In this model of CKD-induced vascular calcification, plasma levels of PTH is suppressed and FGF23 is highly increased due to the treatment with active vitamin D analog (Mace *et al*., JBMR 2021;36:510-522.). Data are expressed as mean±SD. The parameters of the uremic rat were compared to the normal rat, using the two-sided t-test for statistical testing.

**Supplemental Table S3: The media’s calcium concentration in the 8 days mineralization study**

|  | UMR-106 cells | UMR-106 cells +  normal aorta rings | UMR-106 cells +  uremic calcified aorta rings |
| --- | --- | --- | --- |
| Day 1 | 1.24-1.27 mM Ca^2+^ | 1.19-1.27 mM Ca^2+^ | 1.20-1.24 mM Ca^2+^ |
| Day 2 | 1.32-1.35 mM Ca^2+^ | 1.24-1.26 mM Ca^2+^ | 1.22-1.28 mM Ca^2+^ |
| Day 3 | 1.32-1.34 mM Ca^2+^ | 1.25-1.26 mM Ca^2+^ | 1.24-1.26 mM Ca^2+^ |
| Day 4 | 1.27-1.30 mM Ca^2+^ | 1.20-1.22 mM Ca^2+^ | 1.19-1.26 mM Ca^2+^ |
| Day 5 | 1.25-1.27 mM Ca^2+^ | 1.19-1.23 mM Ca^2+^ | 1.18-1.22 mM Ca^2+^ |
| Day 6 | 1.12-1.22 mM Ca^2+^ | 1.17-1.21 mM Ca^2+^ | 1.18-1.23 mM Ca^2+^ |
| Day 7 | 1.17-1.18 mM Ca^2+^ | 1.15-1.18 mM Ca^2+^ | 1.11-1.20 mM Ca^2+^ |
| Day 8 | 0.69-0.71 mM Ca^2+^ | 1.06-1.03 mM Ca^2+^ | 1.06-1.11 mM Ca^2+^ |

The media’s concentration of ionized calcium was measured at actual pH using ABL 900 (Radiometer, Copenhagen, Denmark). The media was collected from three independent experiments. The calcium concentration in the sole media (incubated without cells) was 1.20±0.04.

**Supplemental Figure S1: The osteogenic shift in the uremic calcified aorta**

**
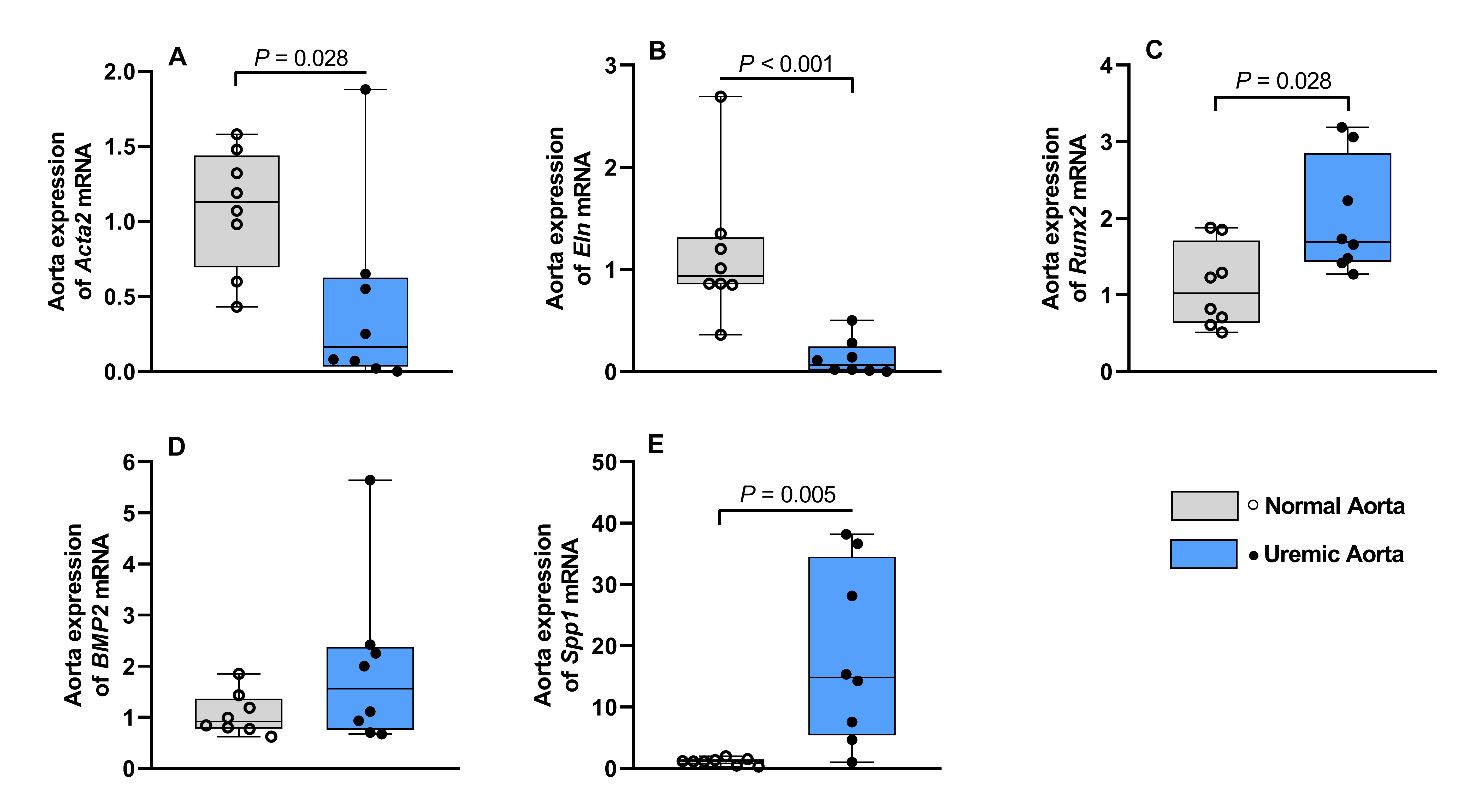
**

**Figure S1****: The osteogenic shift in the calcified aorta from uremic rats.** The aorta from normal rats and the uremic calcified aorta from uremic rats were examined by qPCR. **(A&B)** Vascular smooth muscle cell markers such as α-smooth muscle actin (*Acta2*) and elastin (*Eln*) were significantly downregulated in the calcified aorta from uremic rats**. (C-E)** The uremic calcified aorta was characterized by an osteogenic shift namely the induction of runt-related transcription factor 2 (*Runx2*), bone morphogenetic protein 2 (*BMP2*), and the mineralization inhibitor osteopontin (*Spp1*). All mRNA levels were normalized to the mean of reference genes: *Arbp* and *Rpl13*, and results are shown as the ratio to the mean of the normal aorta expression levels using the ∆∆Ct method. Data are presented in boxplots showing median, interquartile range and all data points. Mann-Whitney U test was used as statistical testing.

**Supplemental Figure S2: Uncropped WB images**

Protein molecular weight marker: Precision Plus Protein kaleidoskope, Bio-Rad, Cat: 161-0375.

**Total β-catenin** (~ 92 kDa, BD Biosciences, 610153)

**
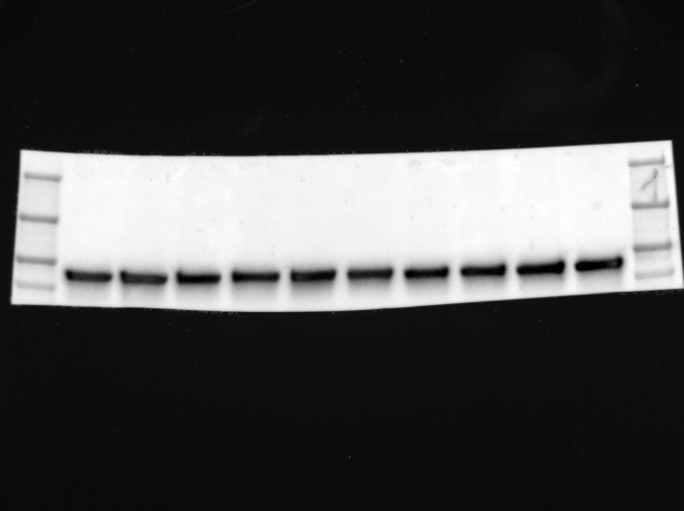
**

**Reference protein** **Park7** (predicted MW 20 kDa, observed bandsize 24 kDa, Abcam, ab18257)


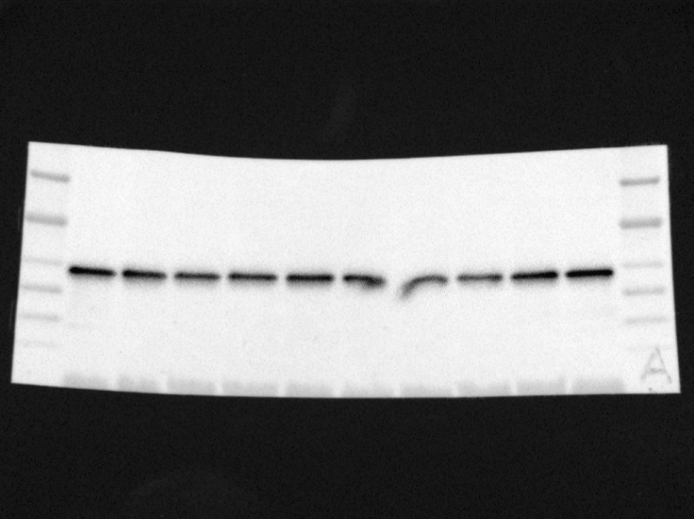


**Active β-catenin** (~ 92 kDa, Merck Millipore, 05-665). To note, a second band was observed.


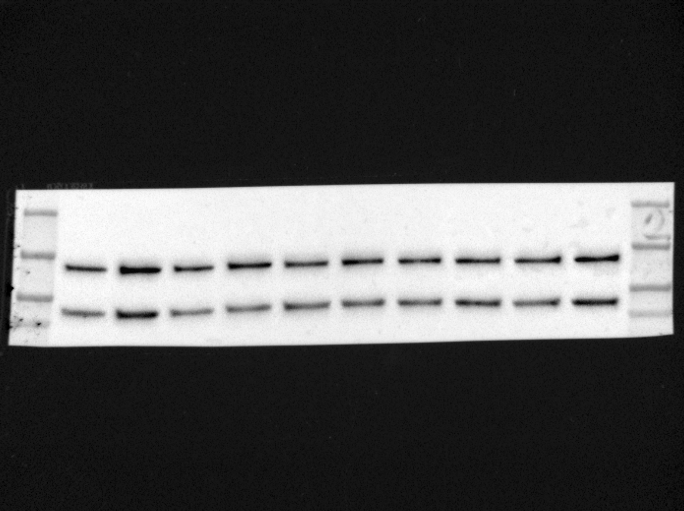


**Reference protein Park7** (predicted MW 20 kDa, observed bandsize 24 kDa, Abcam, ab18257)


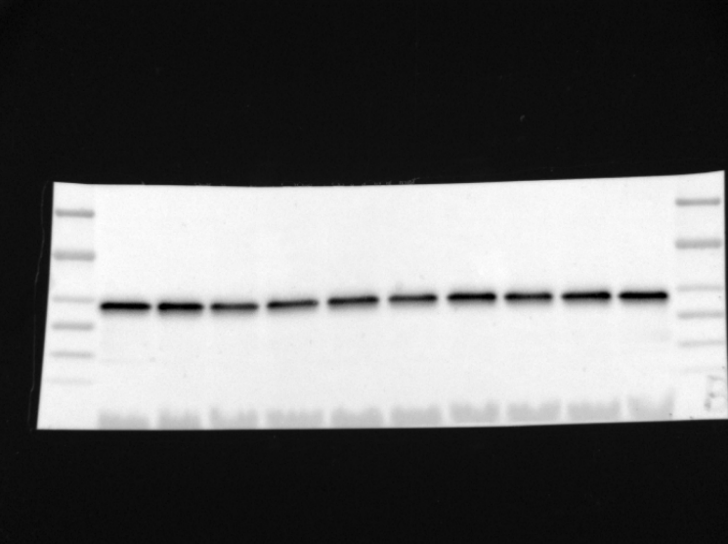


**Osteopontin** (predicted band 66 kDa intact and 32 kDa cleaved, Abcam ab8448).

Only the cleaved protein could be detected.


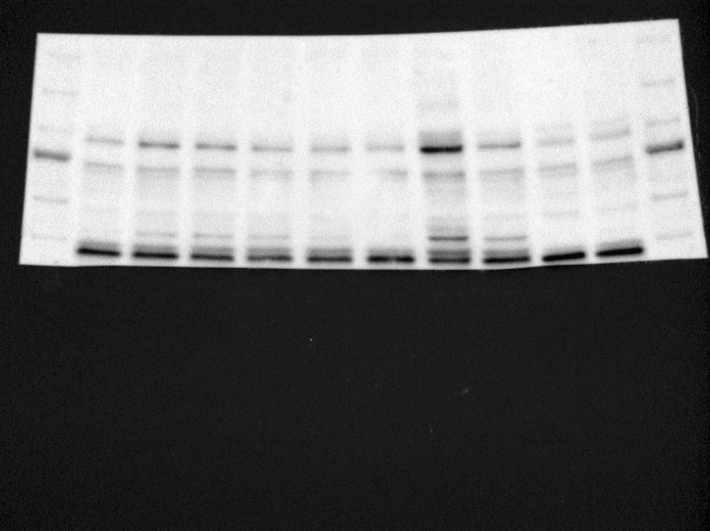


Reference protein Park7 (predicted MW 20 kDa, observed bandsize 24 kDa, Abcam, ab18257)


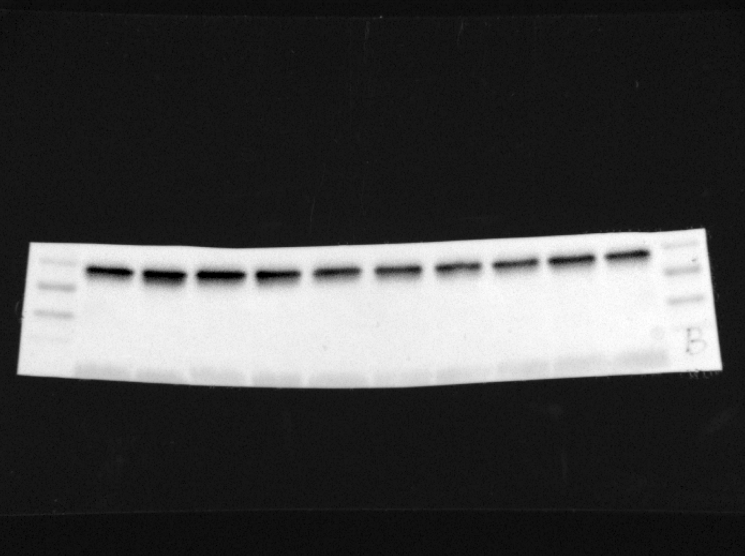


**Supplemental Figure S3: The involvement of canonical Wnt/β-catenin signaling in the vasculature to bone signaling**


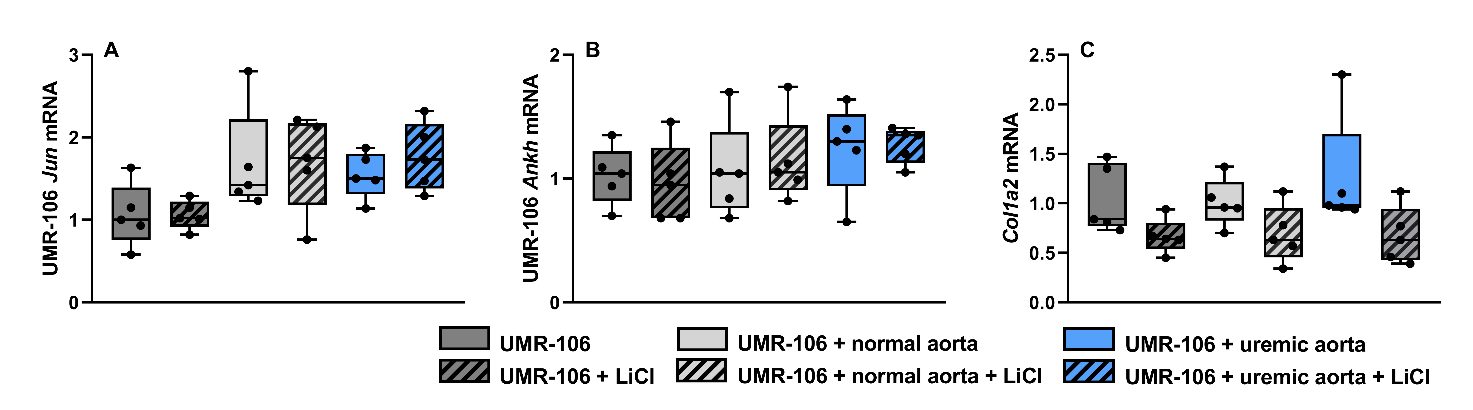


**Figure S3: UMR-106 cells co-incubated with normal and uremic calcified aorta rings with or without lithium chloride (LiCl)**. LiCl stabilizes β-catenin protein by binding GSK-3β, which is part of β-catenin degradation complex, and hereby enhances the canonical Wnt/ β-catenin **(A)** The upregulation of the transcription factor *Jun* by co-incubation with normal and uremic calcified aorta rings was not altered by LiCl. These results indicate that inhibition of the canonical Wnt/β-catenin signaling is not part of this regulation. **(B)** LiCl did not affect the expression of progressive ankylosis protein homolog (ANKH). **(C)** There was a slight trend towards downregulation of collagen I type 2a (*Col1a2*) by LiCl, however, not statistically significant. mRNA levels were normalized to the mean of housekeeping genes, and results are shown as the ratio to the expression level of UMR-106 using the ∆∆Ct method. Data are presented in boxplots showing median, interquartile range and all data points. n=5 in all groups.
